# Successful Retrieval of a Dismembered Central Venous Catheter Stuck to the Right Pulmonary Artery Using a Stepwise Approach

**DOI:** 10.1155/2016/6294263

**Published:** 2016-09-07

**Authors:** Keisuke Nakabayashi, Hidekimi Nomura, Daichi Isomura, Ryo Sugiura, Toshiaki Oka

**Affiliations:** Department of Cardiology, Seirei Hamamatsu General Hospital, Shizuoka, Japan

## Abstract

Recent advances in anticancer chemotherapy have resulted in an increase in the number of patients requiring a central venous port catheter, and the incidence of catheter pinch-off syndrome has been increasing. Catheter pinch-off syndrome is a rare and unusual complication. It is difficult to retrieve dislodged catheters from the pulmonary artery, especially if the catheter is stuck to the peripheral pulmonary artery. We herein describe the successful removal of a catheter stuck in the pulmonary artery with a stepwise approach. First, a pigtail catheter was used to tug the dislodged catheter in order to free the unilateral end. Then, a gooseneck snare was used to catch and pull the catheter out of the patient. The key to success is to free the end of the catheter.

## 1. Introduction

Cancer treatments include the following three main strategies: surgical therapy, chemotherapy, and radiotherapy. Chemotherapy has significantly advanced owing to the development of new anticancer drugs, such as molecularly targeted drugs. However, this approach requires intravenous injection for a long duration, which results in severe vascular pain, inflammation, and the possibility of infection if administered from the peripheral vein. Therefore, cancer patients who receive chemotherapy often receive central venous port catheter (CVPC) implants. However, one complication that can occur with the use of a CVPC is catheter pinch-off syndrome (POS).

POS is caused by intermittent compression of the catheter between the clavicle and first rib and trapping between the subclavian muscle and costoclavicular ligament. This complication is observed in up to 1.0% of patients who have received a CVPC, and 40% of these patients experience catheter dislodgement [1]. Moreover, POS exclusively results from excessive medial venous access of the subclavian vein. The dislodged catheter moves to the superior vena cava, right atrium, right ventricle, and finally pulmonary artery. It is difficult to retrieve dislodged catheters from the pulmonary artery, especially if the catheter is stuck in the peripheral pulmonary artery. In general, prolonged intracardiac manipulation of guidewires and catheters could cause cardiac arrhythmia or cardiac/vascular injury. Therefore, simple and safe maneuvers should be attempted in complicated cases. The key to success is to free the end of the catheter. Here, we report the successful removal of a catheter in the pulmonary artery with a stepwise approach that involved freeing the stuck catheter using a pigtail catheter and catching the catheter using a gooseneck snare.

## 2. Case Presentation

A 40-year-old woman with a mass in her breast and without any specific medical history underwent right muscle-preserving radical mastectomy and level III lymphadenectomy. A pathological study indicated that the mass was an infiltrating duct carcinoma and that there was extranodal invasion. Therefore, she was treated with adjuvant chemotherapy that included adriamycin, cyclophosphamide, paclitaxel, and herceptin; however, after the first course of chemotherapy via the left brachial vein, she experienced peripheral phlebitis of the left brachium and severe nausea. She underwent CVPC implantation surgery at the right subclavian vein using a percutaneous needle puncture approach based on anatomical landmarks without ultrasound guidance. Chemotherapy administration via CVPC was started two months after the implantation. However, infusion resistance and skin swelling on administration of the anticancer drugs via the CVPC appeared nine months after CVPC implantation.

**Figure 1:**
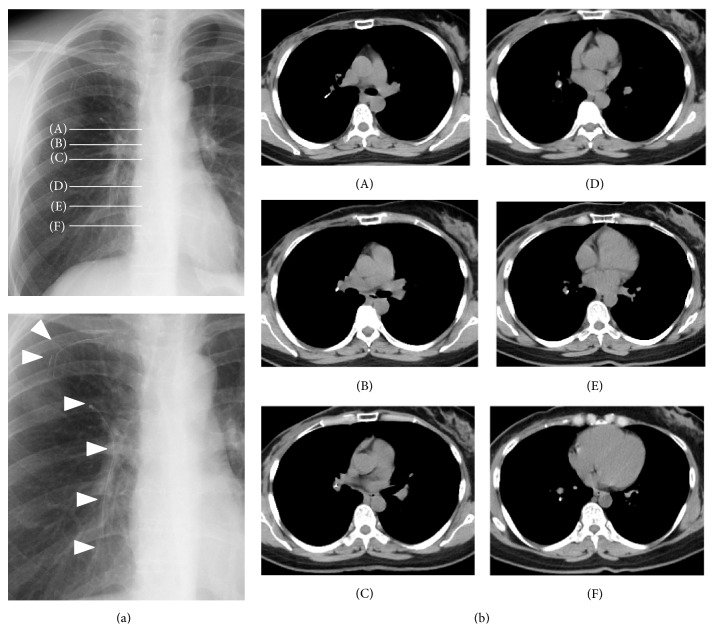
(a) Chest radiographs showing catheter dislodgement due to catheter pinch-off syndrome. Alphabets indicate the slice level of computed tomography. White arrowheads indicate the dislodged catheter and the residual part of the implanted central venous port. (b) Computed tomography images. The dislodged catheter is stuck to the upper and lower pulmonary artery branches.

She was asymptomatic; however, chest radiography revealed that the catheter had dislodged, and computed tomography confirmed that the catheter was stuck to the right pulmonary artery and indicated that it was straddling the right main pulmonary artery (Figure 1). She was referred to our cardiovascular department for removal of the dislodged catheter percutaneously. She agreed to our percutaneous strategy and signed an informed consent form.

**Figure 2:**
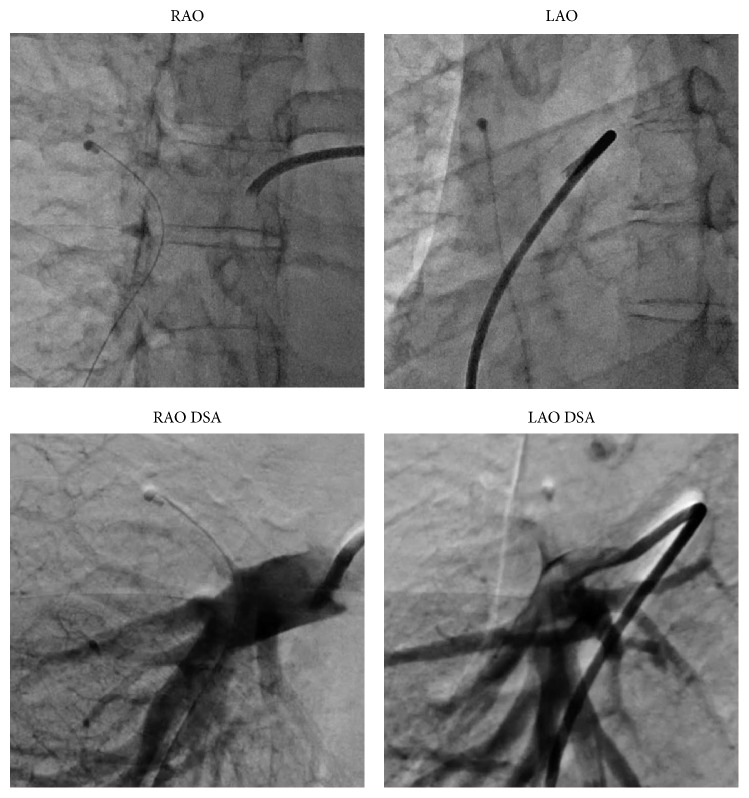
A digital subtraction angiography image showing no flow in the upper pulmonary artery branch, indicating that the dislodged catheter is completely stuck without any gap. RAO: right anterior oblique view; LAO: left anterior oblique view; DSA: digital subtraction angiography.

**Figure 3:**
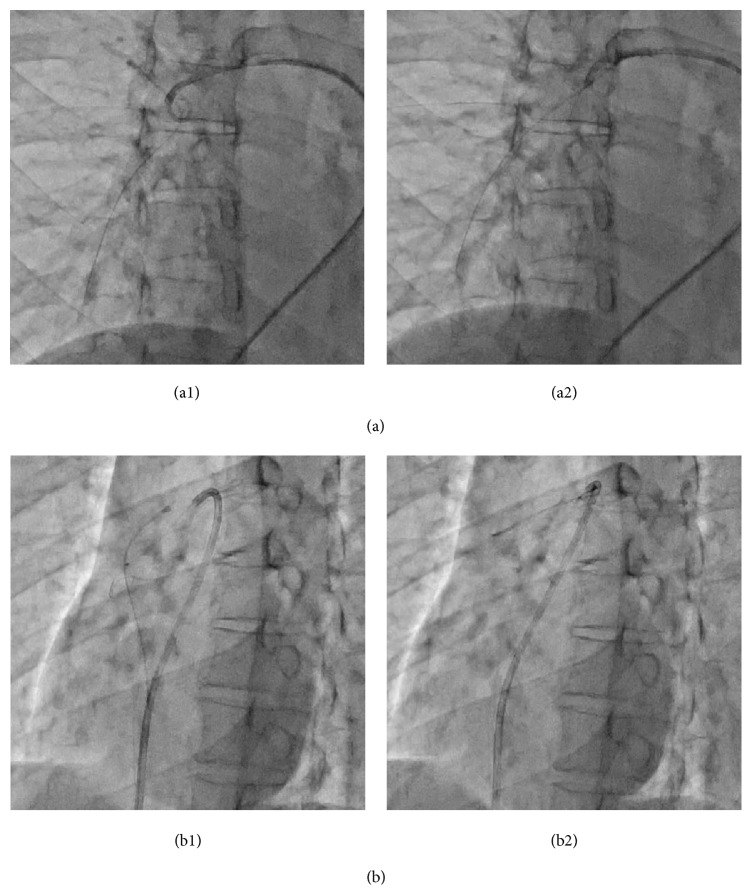
Angiography images. (a1) The pigtail catheter supported by a Judkins Right guiding catheter hooks the dislodged catheter in the bifurcation of the right main pulmonary artery. (a2) The pigtail catheter is pulled and the end of the dislodged catheter is freed in the right main pulmonary artery. (b1) The gooseneck snare catches the body of the dislodged catheter via the free end. (b2) The gooseneck snare is used to pull the dislodged catheter out from the right main pulmonary artery.

We initially inserted an 8-Fr sheath via the right femoral vein. We then administered 5,000 U of heparin after sheath insertion and maintained the activated clotting time between 200 and 300 s. As we performed the procedure immediately after the diagnosis, pre- and postadministration were not performed. Thereafter, we moved an 8-Fr Judkins Right guiding catheter (Mac1, Boston Scientific, Natick, MA, USA) to the right pulmonary artery with a Berman catheter (Harmac Medical Products, Buffalo, NY, USA) and performed pulmonary angiography (Figure 2). One end of the dislodged catheter was stuck to the upper pulmonary artery branch, and the other end was in the lower pulmonary artery branch. Digital subtraction angiography showed no flow in the upper pulmonary artery branch, indicating that the dislodged catheter was completely stuck without any gap. We initially attempted to catch it with a single-loop snare (Amplatz gooseneck snare, Covidien, Dublin, Ireland) and triple-loop snare (EN Snare, Merit Medical Systems, South Jordan, UT, USA); however, we could not grasp the catheter because the acute tapering shape of the pulmonary artery caused difficulty in wire control and the complicated branching prevented the wires from entering the same artery. We then attempted removal with the entwined guidewire technique (crossing multiple guidewires in the same vessel and twisting together to entwine with the dislodged catheter), which is similar to the procedure for retrieving foreign bodies in the coronary artery; however, the procedure failed owing to the same reasons mentioned previously. We realized that selection of the correct distal pulmonary artery was very difficult and that we should use a proximal pulmonary artery. We decided to pull the end of the dislodged catheter from the distal pulmonary artery to the right main pulmonary artery and catch it with a gooseneck snare. We used a pigtail catheter to tug the dislodged catheter. We inserted an 8-Fr Judkins Right guiding catheter near the dislodged catheter, delivered the pigtail catheter straightened with a 0.035-inch guidewire to the distal pulmonary artery, rolled the pigtail catheter by drawing out the 0.035-inch guidewire, and pulled back the pigtail catheter and guiding catheter together. After several attempts, this procedure successfully freed the end of the dislodged catheter in the right main pulmonary artery (Figure 3(a)). Thereafter, we easily grasped the body of the dislodged catheter (Figure 3(b)). As we did not fix the grasping point considering unintended release, the dislodged catheter was replicated during pulling back in the vein. We were unable to place the catheter in the femoral sheath; therefore, we removed the catheter and sheath simultaneously. Hemostasis was easily achieved in a few minutes with manual compression, and no complications were noted.

After removal of the dislodged catheter, the old central venous port was retrieved and a new port was implanted at the right jugular vein to avoid recurrence of catheter POS. The patient has been free from any adverse event for three months. She provided consent for the publication of this case report.

## 3. Discussion

Recent advancements in anticancer treatment have improved the clinical prognosis of cancer patients. As most anticancer chemotherapy protocols require a CVPC, the number of cases of POS has been increasing. This case presents two important clinical requirements. First, all physicians should be familiar with the CVPC and its management. Second, interventionists should be aware of the procedure for retrieval of intravascular foreign bodies percutaneously. The key to success when using a snare is to free the end of the catheter.

A CVPC has been routinely used in cancer patients since the 1980s [2]. This approach has reduced the occurrence of phlebitis, time required for puncture, and chance of infection. However, complications, such as POS, have been reported. POS is caused by the compression of the catheter between the clavicle and first rib, which leads to complete dislodgement (grade 3) [3]. This syndrome can be avoided by using the jugular vein or cephalic vein rather than the subclavian vein. In particular, to avoid excessive medial access of the subclavian vein, the cephalic vein cut-down approach might reduce the complication rate, including the rate of POS [4]. In nursing, it is important to avoid forceful injection and to raise the arm or roll the shoulder during injection to identify positional occlusion earlier [5]. The present case showed moderate resistance to the injected drugs few days prior to recognition; therefore, we might be able to avoid this complication prior to complete dislodgment.

If we cannot identify POS in advance and the dislodged catheter enters the right cardiac system, percutaneous retrieval should be attempted first owing to its high success rate and minimal morbidity. Pulmonary embolization of the dislodged catheter might increase the risk of possible complications, such as thrombus formation, which can lead to pulmonary embolism and infection [6]. Although gooseneck loop snares, such as the one used in the present case, are popular for endovascular retrieval of dislodged catheters, they cannot be used if the end of the catheter is not accessible. In such situations, basket snares may be more appropriate or the end of the dislodged catheter should be freed. Additionally, pigtail or Simmons catheters may be used to reposition the dislodged catheter in order to facilitate capture. In this procedure, it is important to introduce the dislodged catheter into the inferior vena cava rather than the pulmonary artery or right ventricle by using the rolling pigtail catheter technique in order to avoid cardiac or vascular complications. We attempted this approach; however, it was technically difficult. Other options include bronchoscopic forceps, myocardial biopsy catheters, Fogarty catheters, hook guidewires [7], ablation catheters [8], and inflated coronary balloons [9]. Considering medical economics and safety, pigtail catheters are the most suitable, and they are familiar to all interventionists (Table 1). It is appropriate to divide the procedure into several steps when the situation is complicated. The gooseneck snare generally does not work appropriately in a large vessel. In our case, the gooseneck snare fortunately caught the dislodged catheter in the right main pulmonary artery. If we had not caught the dislodged catheter with the gooseneck snare, we would have used a homemade big-loop snare [10].

There are some safety concerns. We could not perform pulmonary angiography after the procedure to confirm patency, because we had to retrieve the dislodged catheter and guiding catheter simultaneously. As this procedure might cause pulmonary artery injury, we strongly recommend pulmonary angiography or contrast computed tomography immediately after the procedure, if possible. In addition, this patient was an outpatient, and this procedure could be performed on an outpatient basis. However, we believe that inpatient management is better from the point of view of safety (i.e., hemostasis, arrhythmia monitoring, subacute pulmonary embolization, and vascular injury).

We reported the successful removal of a catheter stuck in the pulmonary artery with a stepwise approach that involved freeing the catheter using a pigtail catheter and catching the catheter using a gooseneck snare. POS is relatively rare; however, it is a dangerous complication and its incidence is increasing with the increase in the use of CVPCs. Cardiovascular interventionists are generally unfamiliar with malignant diseases and CVPCs; however, if POS occurs, the situation should be appropriately handled. The key to success is to free the end of the catheter.

In summary, catheter pinch-off syndrome is a rare and unusual complication. It is difficult to retrieve dislodged catheters from the pulmonary artery, especially if the catheter is stuck to the peripheral pulmonary artery. We herein describe the successful removal of a catheter stuck to the pulmonary artery using a stepwise approach. First, a pigtail catheter was used to tug the dislodged catheter in order to free the unilateral end. Then, a gooseneck snare was used to catch and pull the catheter out of the patient. The key to success is to free the end of the catheter.

## Figures and Tables

**Table 1:** Various materials used to retrieve foreign bodies reported in the literature.

Authors	Material (type)	Merits Demerits	Cost in Japan (US$)
Önal et al. [11]	Pigtail catheters (RD)	Very low cost, low risk of vessel injury Limited range of motion	20
Kawata et al. [8]	Ablation catheters (RD)	Intentional movement Very high cost, risk of vessel injury	1,300
Yedlicka Jr. et al. [12]	Gooseneck snares (CD)	Easy availability Need for a free end, not a strong grip	400
Kawata et al. [8]	Basket snares (CD)	Adjustment of vessel size, relatively strong grip Risk of vessel injury	400
Fisher and Ferreyro [7]	Forceps (CD)	Very strong grip Risk of vessel injury, high cost	820
Schricker et al. [9]	Coronary balloons (CD)	Applicability to tubular foreign bodies Need for a guidewire, risk of vessel injury	540

Cost is calculated at the exchange rate of 110 yen to 1 US dollar.

RD: repositioning device; CD: catching device.
